# Optimizing Instruction Scheduling and Register Allocation for Register-File-Connected Clustered VLIW Architectures

**DOI:** 10.1155/2013/913038

**Published:** 2013-07-18

**Authors:** Haijing Tang, Xu Yang, Siye Wang, Yanjun Zhang

**Affiliations:** ^1^School of Software, Beijing Institute of Technology, Beijing, China; ^2^School of Information and Electronics, Beijing Institute of Technology, Beijing, China

## Abstract

Clustering has become a common trend in very long instruction words (VLIW) architecture to solve the problem of area, energy consumption, and design complexity. Register-file-connected clustered (RFCC) VLIW architecture uses the mechanism of global register file to accomplish the inter-cluster data communications, thus eliminating the performance and energy consumption penalty caused by explicit inter-cluster data move operations in traditional bus-connected clustered (BCC) VLIW architecture. However, the limit number of access ports to the global register file has become an issue which must be well addressed; otherwise the performance and energy consumption would be harmed. In this paper, we presented compiler optimization techniques for an RFCC VLIW architecture called Lily, which is designed for encryption systems. These techniques aim at optimizing performance and energy consumption for Lily architecture, through appropriate manipulation of the code generation process to maintain a better management of the accesses to the global register file. All the techniques have been implemented and evaluated. The result shows that our techniques can significantly reduce the penalty of performance and energy consumption due to access port limitation of global register file.

## 1. Introduction

very long instruction words (VLIW) architecture [1] typically has multiple functional units (FUs), which allows multiple instructions to be executed in parallel. This feature offers a significant opportunity to enhance the instruction level parallelism (ILP), also largely enhancing the processing ability, which is very desirable in encryption application domain. However, if centralized register file is used, when the number of FUs in the VLIW architecture grows large, there will be a strong pressure on the register file.

First, the number of needed registers becomes huge, when the number of FUs grows large. Typically, large register file is area consuming and will lead to more energy consumption.

Second, the number of accesses to the register file becomes huge, when the number of FUs grows large, either read or write. This will unavoidably lead to access conflicts when there are no sufficient access ports to the register file. Some of the FUs might need to wait until others finish accessing the register file. This will lead to performance degradation and more energy consumption. The problem is that we cannot solve it by simply increasing the number of access ports to the register file because that will both increase the design complexity of the register file, and lead to significant growth in area and energy consumption of the register file.

So, clustering becomes a common trend in the design of VLIW architecture due to its ability to alleviate power-, thermal-, and complexity-related problems of unclustered VLIW architecture.

In a clustered VLIW architecture, the FUs and register files are divided into several smaller groups. Each group is called a cluster. FUs can directly access data stored in registers of its own cluster. However, inter-cluster data access needs some specific mechanism.

Traditional clustered VLIW architectures use buses to connect different clusters. In the bus-connected clustered VLIW (BCC VLIW) architecture, when an inter-cluster data communication occurs, an explicit data moving instruction is inserted in the original instruction queue. The data moving instruction accesses data stored in the remote cluster and moves it to one of the registers in the local register file. The execution of this additional data moving instruction needs resources, consumes additional energy, and has nonzero latency. The insertion of these additional data moving instructions might lead to extension of total execution time, which in turn might cause performance degradation, and an increase of energy consumption.

Register-file connected clustered VLIW (RFCC VLIW) architecture has been developed to overcome this performance and energy consumption penalty related to BCC VLIW architecture [2]. In RFCC VLIW architecture, local register file of each cluster can only be accessed by the FUs in that cluster, same mechanism as in BCC VLIW architecture. The difference is that there is also a global register file in RFCC VLIW, which can be accessed by all the FUs through the access ports of its own cluster, either read or write. So, when an inter-cluster data communication is needed, the FU which generates the data writes it in the global register file, and the FU needing that data reads the data from the global register file.

Compared to the BCC VLIW architecture, the advantages of RFCC VLIW architecture are (1) zero latency for inter-cluster data communications; (2) no need for additional inter-cluster data moving instruction. Thus, using RFCC VLIW architecture can avoid performance degradation and energy penalty due to inter-cluster data moving instruction as in BCC VLIW.

However, for the consideration of design complexity, area, and energy efficiency of the global register file, the number of access ports to the global register file from each cluster should be limited. Thus, the accesses to the global register file must be well managed; otherwise, there will be conflicts when the number of simultaneously accesses to the global register file exceeds the number of access ports. The conflicts lead to delay of some accesses to the global register file, which means the delay of execution of some instructions. This may lead to the extension of the whole execution time, which means performance degradation and more energy consumption.

So, we need to minimize the situation where access conflicts to the global register file happen for RFCC VLIW architecture, for the sake of performance enhancement and energy consumption reduction.

The problem can be solved by (1) minimizing the number of accesses to global register file or (2) balancing the distribution of the access to global register file among the whole execution time so as to minimize the situation where the number of simultaneously accesses exceeds the port limitation. And, in order to minimize the number of accesses to global register file, we could (1) minimize unnecessary inter-cluster data communications and (2) minimize unnecessary global register allocation.

The main contributions of this work are (1) force-balanced-two-phase (FBTP) instruction scheduling algorithm to minimize unnecessary inter-cluster data communications and balance the distribution of the access to global register file among the whole execution time; (2) localization-enhanced (LE) register allocation mechanism to minimize unnecessary global register allocation.

The Lily architecture is of RFCC VLIW architecture. It is designed for real-time video encryption system, which demands high performance and low energy consumption at the same time. We have implemented the presented techniques in LilyCC compiler designed for Lily architecture.

This paper is organized as follows: Section 2 will discuss the Lily architecture; in Section 3, we will give an introduction to LilyCC compiler; the FBTP instruction scheduling algorithm is presented in Section 4; Section 5 describes LE register allocation mechanism for RFCC VLIW architecture; related works will be discussed in Section 6; we will discuss the experimental framework and results in Section 7; and finally we give conclusions in Section 8.

## 2. Architecture of Lily

The details of the Lily architecture can be found in [3], so we only give a brief description here. The Lily architecture is a scalable RFCC VLIW architecture. The scalability includes the number of cluster, the number and type of FUs in each cluster, the number and width of registers in the local register file, the number and width of registers in the global register file, the number of read and write access ports to the global register file of each cluster, and the instruction set.

The Lily architecture is dedicated for fixed-point processing, and does not support float-point processing. There are three different types of FUs presented in current design, which are Unit A, Unit M, and Unit D, respectively. Unit A can execute arithmetic instructions, logical instructions, and shift instructions. Unit M can execute multiplication instructions, as well as some arithmetic and logical instructions. Unit D is in charge of memory access and process controlling and can execute some arithmetic and logical instructions.

The Lily architecture has a combined instruction set of both 16-bit instructions and 32-bit instructions, to provide better flexibility. They can be distinguished by the second and third least significant bits of the instruction code. Designer using Lily architecture can customize their own instruction set by choosing instructions from the default instruction set. Lily instruction set includes specific instructions for speeding up the multimedia signal processing, instruction dedicated for encryption operation, and SIMD instructions.

**Figure 1:**
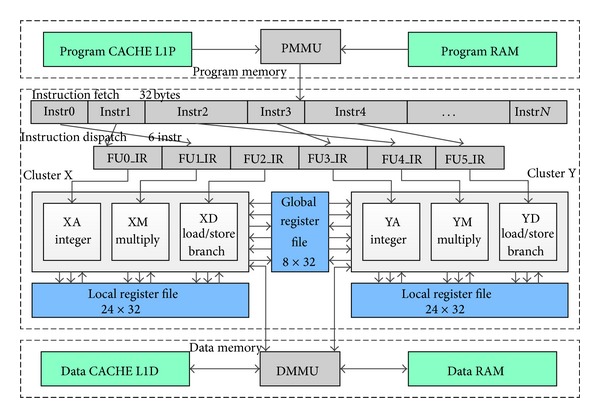
An example of the Lily architecture.

An example of Lily architecture is shown in Figure 1. It has two clusters, and there are three FUs in each cluster, one of each type. Each cluster has its own local register file, composed of 24 registers of 32 bits. The global register file consists of 8 registers of 32 bits. There are two read access ports and one write access port to the global register file from each cluster.

There are 4 bits in the instruction code of the 16-bit instruction dedicated to register access, so they can access only 16 registers. So, in this example, 16-bit instruction can access only 4 of 8 global registers, and 12 of 24 local registers 32-bit instruction has 5 bits for register access, so they can access all the 8 global registers and the 24 local registers in this case.

## 3. LilyCC Compiler

**Figure 2:**
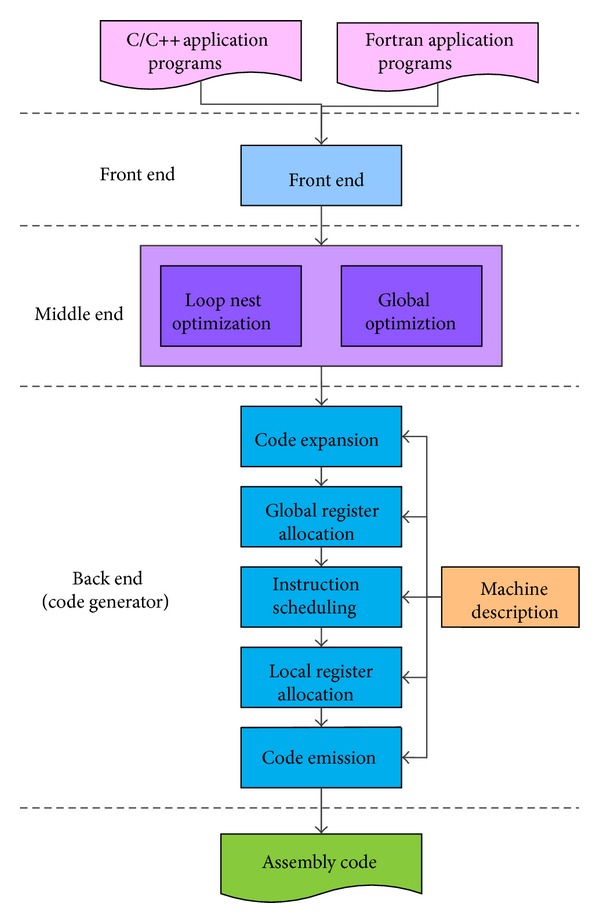
Structure of LilyCC compiler.

LilyCC [3] is designed based on Open64 compiler. The architecture of LilyCC is illustrated in Figure 2. In LilyCC, we have implemented four different optimization levels, which are O0, O1, O2, and O3, respectively.

 LilyCC is composed of three parts. The front end takes application programs written in C/C++/Fortran languages as input, performs syntax and semantics checking and analysis, and translates the application programs into intermediate representation (IR) structures. The middle end contains optimization phases like loop nest optimization, global optimization, and so on. The back end, or the code generator, translates the IR structure into final assembly code and emits them. Many target-dependent optimization phases, including control flow optimization, extended block optimization, and software pipelining, are performed in the back end.

LilyCC compiler is retargetable. The information of hardware architecture is stored in the machine description files. To retarget to a new architecture, the machine description files must be implemented first. The machine description files contain information of the instruction set architecture (ISA), the application binary interface (ABI), and the processor model of the target architecture.

The code generator of LilyCC can be divided into five major phases: code expansion, global register allocation, instruction scheduling, local register allocation, and code emission.

LilyCC compiler supports automatic vectorization. A lot of existing approaches in research perform automatic vectorization at a late stage of the compilation process, that is, in the back end, because more information is available at the back end, such as a more precise data flow of the input program and the info about the underling target hardware. However, the disadvantage is that the data parallelism in loops cannot be effectively exploited by these techniques, so the code quality can be less optimal.

The automatic vectorization technique used in LilyCC is similar as the one used in [4], which is a high-level automatic vectorization technique to generate vectorized code by examining the loop code. It runs in the early stage of the compilation process, just after the input source code program has been transformed into the IR structure. As this approach only needs simple knowledge of the target machine's instruction set architecture, it is easily retargetable.

## 4. FBTP Instruction Scheduling Algorithm

In order to enhance performance and energy efficiency, instruction scheduling process for RFCC VLIW architecture has three tasks: (1) minimizing the number of inter-cluster data communications; (2) balancing the distribution of inter-cluster data communications to minimize the situation where the number of concurrent inter-cluster data communications exceeds the number of registers in the global register file or the number of read or write ports to the global register file from one cluster at a single clock cycle; (3) minimizing the number of execution cycles.

In FBTP instruction scheduling algorithm, the three tasks are achieved by the following.

1. Dividing the instruction scheduling process into two phases: Predecision phase and main scheduling phase. The first phase outputs a preliminary cluster assignment decision for all the instructions. The second phase performs cycle scheduling according to the cluster assignment decisions from the first phase. Although the decisions of cycle scheduling and cluster assignment are made in separate phases, the main interactions between cluster assignment and cycle scheduling are actually estimated and considered.
2. Using gravitation force (GF) Array to describe the data dependence relations between instructions, and using repulsion force (RF) Array to describe the resource availability. The two forces are balanced to conduct the cycle scheduling and cluster assignment, so as to minimize the number of inter-cluster data communications and the number of execution cycles.
3. Transforming the distribution of inter-cluster data communications into data dependence relations between instructions and resource availability, when calculating GF array and RF array, in order to minimize the number of concurrent inter-cluster data communications.

### 4.1. The Predecision Phase

The procedure of Predecision phase is shown in Algorithm 1. The input of the Predecision phase is the Data Dependence Graph (DDG). DDG can be denoted as DDG = {*N*, *E*}, where *N* is the set of instructions in DDG and *E* is the set of edges in DDG. In Predecision phase, all the instructions will be prescheduled to a *Schedule-Point *(*p*, *q*), where *p* denotes the cluster, and *q* denotes the clock cycle. The cluster assignment decision for all the instructions is the output of the Pre-Decision phase, while the clock cycle pre-scheduled for each instruction is used only in this phase for estimating and considering the interactions between cluster assign and cycle schedule.

As soon as Possible (ASAP) scheduling and as late as possible (ALAP) scheduling are performed to get the earliest possible execution cycle *T*_*e*_ and the latest possible Execution Cycle *T*_*l*_ for each instruction in the ready list. Then an instruction is selected from the ready list according to predefined rules.

Gravitation force (GF) values and repulsion force (RF) values are calculated for that instruction at every possible schedule point. Then the GF values and RF values are normalized to calculate the Balance Force (BF) values. The algorithm finds out the schedule point with the maximize BF value, and schedules the instruction to it.

The process is repeated until all the instructions are successfully pre-scheduled. The details of this algorithm will be discussed in the following.

#### 4.1.1. Calculation of GF Value

Gravitation force value GF(*i*, *x*, *y*) indicates the tightness of data dependence relation between *Instruction i* and *Schedule-Point *(*x*, *y*). The calculation of GF values only applies to the possible schedule point of *Instruction i*.

There are three factors that will influence the GF value.

- The number of data dependence relations from each cluster. For the purpose of minimizing the number of inter-cluster data communications, we would like instructions having data dependence relations to be placed in the same cluster. For example, when *Instruction i* is to be prescheduled, if there are three data dependence relations from *Cluster A* and only one data dependence relation from *Cluster B*, then, assigning *Instruction i* to *Cluster A* would be a better choice, because we only have one inter-cluster data communications.
- The span of the data dependence relations. If the number of active inter-cluster data communications exceeds the number of registers in the global register file, then some instructions must delay their write access to the global register file. So, if an inter-cluster data communication is unavoidable, then we would like it to be a short one. For example, if both *Instruction j* from *Cluster A* and *Instruction k* from *Cluster B* have data dependence relations with *Instruction i* and *Instruction j* is scheduled two clock cycles before *Instruction k*, then when *Instruction i* is to be prescheduled, it is preferred to pre-schedule *Instruction i* to *Cluster A*, because in that case, we will get a shorter inter-cluster data communications.
- The number of active inter-cluster data communications at *Schedule-Point *(*x*, *y*) of instructions from the neighborhood of *Instruction i*. the neighborhood of *Instruction i*, *B*(*i*) is defined as the set of instructions that have data dependence relations with *Instruction i*. And an active inter-cluster data communication from *Instruction j* means that (1) *Instruction j* is not in *Cluster x* (2) the inter-cluster data communication from *Instruction j* goes to *Cluster x*, and (3) the inter-cluster data communication is not finished at *Cycle y*.

When calculating gravitation force, these three factors must all be taken into consideration.

In Step 5 of Algorithm 1, *δ*(*j*, *x*) denotes the possibility that *Instruction j* from the neighborhood of *Instruction i* is in *Cluster x*. It is mainly used to estimate the influence of the number of data dependence relations from each cluster on GF value. *W*(*i*, *j*, *x*, *y*) denotes the weight of the edge between *Instruction i* and *Instruction j*, which is defined as the span of that edge. It is used to estimate the influence of the second factor on GF value. *t*(*j*) is the execution time of *Instruction j*. *N*_*c*_(*j*) denotes the number of clusters that *Instruction j* can be scheduled in. *λ*(*j*, *x*, *y*) is the number of active inter-cluster data communications from *Instruction j*, which is a member of the neighborhood of *Instruction i*, to *Cluster x* at *Cycle y*. It is mainly used to estimate the influence of the third factor on GF value.

#### 4.1.2. Calculation of RF Value

Repulsion force value RF(*i*, *x*, *y*) represents the resource availability when *Instruction i* is to be prescheduled to *Schedule-Point *(*x*, *y*). There are two factors that will influence the RF value.

- The available resources in each cluster. For the purpose of minimizing the number of execution cycles, we need to distribute instructions evenly in each cluster, which means we would like to pre-schedule instructions to cluster which has more available resources.
- The existed inter-cluster data communications in each cluster. As we know, for the purpose of balance the distribution of inter-cluster data communications, it is beneficial to pre-schedule instructions to cluster which has smaller number of existed inter-cluster data communications.

In step 6 of Algorithm 1, *M*(*j*) is the mobility of *Instruction j*, which indicates the possibility of *Instruction i* to move between different cycles. *γ*(*j*, *x*, *y*) denotes the possibility that *Instruction j* is in *Schedule-Point *(*x*, *y*). ∑_*j*_ *γ*(*j*, *x*, *y*) represents current resource occupation at *Schedule-Point *(*x*, *y*). It is used to calculate the influence of the first factor on RF value. *β*(*x*, *y*) is the number of existed active inter-cluster data communications from other clusters to *Cluster x* at *Cycle y*. It is used to calculate the influence of the second factor on RF value.

#### 4.1.3. Calculation of BF Value

As discussed before, instruction scheduling process for RFCC VLIW architecture has three tasks: (1) minimizing the number of inter-cluster data communications; (2) balancing the distribution of inter-cluster data communications to minimize the situation where the number of concurrent inter-cluster data communications exceeds the number of registers in the global register file or the number of read or write ports to the global register file from one cluster at a single clock cycle; (3) minimizing the number of execution cycle.

In order to fulfill the first task, the instruction should be prescheduled to the schedule point that has the largest GF value. For the third task, the instruction should be prescheduled to the schedule point with the least RF value. And for the second task, we would like to schedule instruction to the schedule point with the largest GF value and the least RF value. So, we should take into account both GF and RF values.

Thus, we have introduced balance force (BF) to comprehensively consider the influence of both GF values and RF values. In order to calculate BF values, both GF values and RF values of all the possible schedule points must be normalized first. Then the BF values is calculated as indicated in step 8 of Algorithm 1.

### 4.2. Main Scheduling Phase

After the Predecision phase, the preliminary decision of cluster assignment of all the instructions is delivered to the second phase. The main scheduling phase is a modified version of the list-scheduling algorithm [5]. The commonly used heuristics—scheduling those instructions on the critical path first—is used to guide the selection order of instructions from the ready list. Here, instruction with mobility of zero is defined as on the critical path.

As the set of instructions with mobility of 0 would change dynamically during the schedule process, thus in order to greedily ensure that instructions with mobility of 0 always be selected first, after scheduling of each instruction, the algorithm must update the earliest possible execution cycle and the latest possible execution cycle for all the unprocessed instructions. This could guarantee that the stretching of critical paths is minimal and subject to the finite resource constraints of target machine.

### 4.3. Complexity of the FBTP Algorithm

In FBTP algorithm, let *n* be the number of instructions. For each instruction, it will take at most *O*(*n*) effort to calculate the GF value and at most *O*(*n*) effort to calculate the RF value. Thus, it will take at most *O*(2*n* ^2^) effort to finish the Predecision stage. In the main scheduling, it will take at most *O*(*n*) effort to finish the cycle scheduling procedure.

Thus the worst-case complexity of FBTP algorithm is *O*(*n* ^2^), whereas the worst-case complexity of list schedule is *O*(*n* ^2^log⁡*n*).

## 5. Localization-Enhanced Register Allocation Mechanism

The localization-enhanced (LE) register allocation mechanism for RFCC VLIW architecture is presented in Algorithm 2. It is used as an enhancement engine for register allocation in basic block (BB). The main purpose of this mechanism is to guide the register allocation process so as to avoid unnecessary allocation of global register. In this mechanism, we guarantee that only two kinds of variables have the privilege to be allocated to the global register: (1) the variables active at the exit of a BB, to provide generality; (2) the variables of which their def and uses have different clusters.

Let *n* be the number of instructions. Then the worst-case complexity of LE mechanism is *O*(*n*).

## 6. Related Work

Since the introduction of VLIW [1] in 1983, there have been many researches reported. Payá-Vayá et al. [6] have presented a forwarding-based approach to increase the code compaction of VLIW media processors, so as to enhance the performance and to reduce the number of needed read/write ports to the register file. Wang and Chen [7] have introduced an architecture-dependent register allocation and instruction scheduling algorithm for VLIW architecture. Uchida et al. [8] have present an energy-aware SA-based instruction scheduling for fine-gained power-gated VLIW processors.

As clustering has become a common trend, there emerged a lot of works concerning either the instruction scheduling or the register allocation of clustered architectures.

Zalamea et al. [9] have presented an instruction scheduling, algorithm for clustered VLIW architecture, which uses limited backtracking to reconsider previously taken decisions, thus providing the algorithm with additional possibilities for obtaining high throughput schedules with low spill code requirements. Codina et al. [10] have introduced a modulo scheduling framework for clustered ILP processors that integrates the cluster assignment, instruction scheduling, and register allocation steps in a single phase. The proposed framework includes a mechanism to insert spill code on the fly and heuristics to evaluate the quality of partial schedules considering simultaneously inter-cluster communications, memory pressure, and register pressure. Later, they have exploited a concept of virtual cluster to assist the instruction scheduling for clustered architecture [11].

In 2001, Aleta et al. [12] have presented a graph-partitioning-based instruction scheduling for clustered architecture. In 2009, they [13] have presented another graph-based approach, called AGAMOS, to modulo-schedule loops on clustered architectures, which uses a multilevel graph partitioning strategy to distribute the workload among clusters and reduces the number of inter-cluster communications at the same time. Arafath and Ajayan [14] have implemented an integrated instruction partitioning and scheduling technique for clustered VLIW architectures, which is a modified list scheduling algorithm using the amount of clock cycles followed by each instruction and the number of successors of an instruction to prioritize the instructions. Zhang et al. [15] presented a phase coupled priority-based heuristic scheduling algorithm, which converts the instruction scheduling problem into the problem of scheduling a set of instructions with a common deadline.

Xu et al. [16] have presented their study on the design of inter-cluster connection network in clustered DSP processors. The approach starts with determining the minimum number of buses required in polynomial time for any given schedules and then further determines an underlying inter-cluster connection scheme with the number of buses determined in the previous step. They have also given a computation and communication coscheduling algorithm to generate schedules which lead to fewer minimum buses required for the inter-cluster connection network. Nagpal and Srikant [17] have presented their instruction scheduling algorithm which exploits the limited snooping capability of snooping-based clustered VLIW architectures to reduce the register file energy consumption.

Huang et al. [18] have introduced a worst-case-execution-time-aware re-scheduling register allocation (WRRA) approach, which is used to achieve worst-case-execution-time (WCET) minimization for real-time embedded systems with clustered VLIW architecture. In this approach, the effects of register allocation, instruction scheduling, and cluster assignment on the quality of generated code are all taken into account for WCET minimization. Yang et al. [19] have presented a triple-step data-dependence-graph-based (TDB) scheme for clustered VLIW architecture, which performed a backtracking optimization after instruction schedule to bring further improvement.

However, these researches are all focused on BCC VLIW architecture. The efforts focusing on the optimization for RFCC VLIW architecture are not much.

Zhou et al. [20] have presented a two-dimension force-directed (TDFD) scheduling algorithm for RFCC VLIW architecture. It is used as the default instruction scheduling algorithm in LilyCC compiler. However, TDFD simply considered the balancing of influences of data dependence relations and available resources on instruction scheduling, but has not actually taken into account the influence of limitation on access ports to the global register file on the instruction scheduling.

## 7. Results and Discussions

### 7.1. Experimental Framework

To evaluate the effectiveness of our algorithm, we used a suite of 20 applications from different benchmark sets. The characteristics of these application codes can be found in [21, 22]. The domain we focused on is the multimedia processing, which depends heavily on the capability to perform DSP applications. We chose these applications for their qualified representative in the DSP scope.

All analyzed benchmarks were validated against precompiled binaries in the original benchmark suite. We have built a simulator for Lily architecture, based on Gem5 [23] simulator. This simulator is used to run the compiled benchmarks and to collect data. The energy model used in our simulator is based on [24]. We have conducted a series of RTL simulations, using Cadence EDA tool chain to extract the parameters needed for construction of the energy model.

The effectiveness of our proposed techniques are compared with several state-of-the-art techniques, including TDFD [20] (LilyCC's default instruction scheduling algorithm), AGAMOS [13], and TDB [19] algorithms.

### 7.2. Results and Discussions

#### 7.2.1. Evaluation of the Influence of the Number of Global Registers on Performance and Energy Consumption

**Figure 3:**
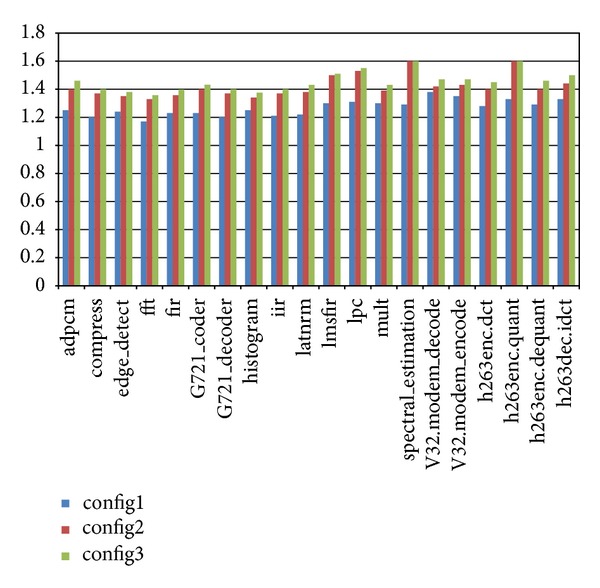
Evaluation of performance with different scales of global register file.

In order to evaluate the influence of the number of global registers, we have defined three configurations. All the three configurations have two clusters. Each cluster has one Unit A, one Unit M, and one Unit D. And there are 2 read ports and 1 write port to the global register file for each cluster. The first configuration has 4 global registers in the global register file, the second one has 8 global registers, and the third one has 16 global registers.  Figure 3 shows the evaluation of performance when the scale of global register file varies. The result shown in the figure is the performance enhancement with respect to the default LilyCC scheduler TDFD.

In all the situations, FBTP outperforms TDFD. The performance enhancement for the first configuration is in general less than the other two configurations. The reason is that, in the first configuration, the number of global registers is too small. So the chance that global register access conflicts happen is high, and there is not much space for optimization.

**Figure 4:**
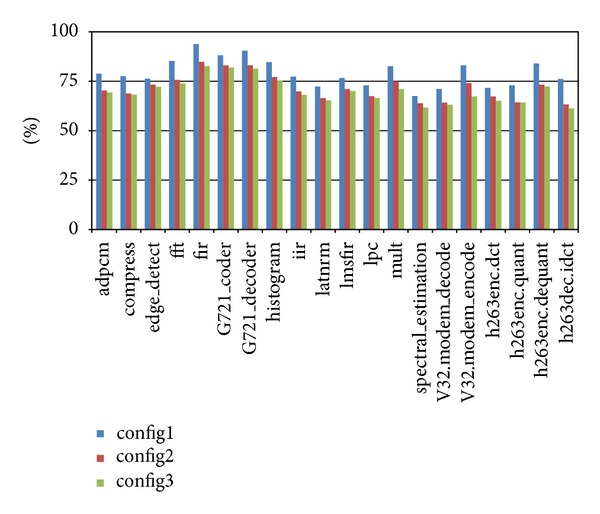
Evaluation of energy consumption with different scales of global register file.

And it can also be noticed that the differences between the performance enhancement for configuration 2 and for configuration 3 are not much. Actually, if the number of global registers is larger than the maximum number of possible concurrent inter-cluster data communications, there will be no extra gain. The results of comparison of energy consumption shown in Figure 4 also indicate this.

#### 7.2.2. Evaluation of the Influence of the Number of Access Ports to the Global Register File on Performance and Energy Consumption

When evaluating the influence of the number of access ports to the global register file on performance and energy consumption, we choose 4 configurations. All the configurations have 2 clusters. Each cluster is composed of one Unit A, one Unit M, and one Unit D. There are 8 registers in the global register file. The first configuration has 1 read port and 1 write port in each cluster. The second configuration has 2 read ports and 1 write port in each cluster. The third configuration has 3 read ports and 2 write ports in each cluster. The fourth configuration has 4 read ports and 2 write ports in each cluster.

**Figure 5:**
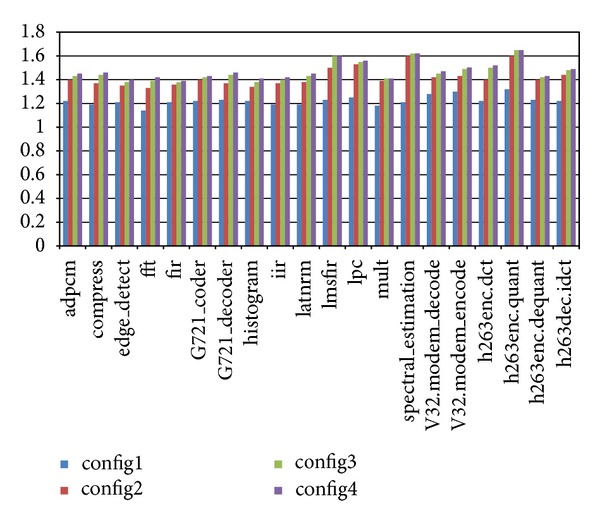
Evaluation of performance with different configurations of access ports.

The comparison of performance enhancement with respect to TDFD is shown in Figure 5. From the picture, we can see that when the number of access ports to the global register file grows, the performance enhancement improves. However, the area, design complexity, and energy consumption cost related to the access ports must also be taken into consideration when designing the processor. The differences of performance enhancement between configuration 3 and 4 are much smaller compared to the differences between configuration 1 and 2. So, configuring each cluster of 2 read ports and 1 write port might be a reasonable choice.

**Figure 6:**
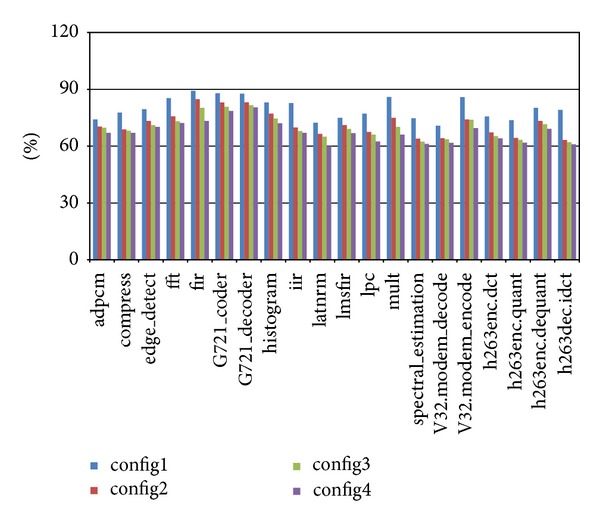
Evaluation of energy consumption with different configurations of access ports.

The energy consumption of FBTP compared with TDFD is shown in Figure 6.

#### 7.2.3. Evaluation of the Influence of the Number of Clusters on Performance and Energy Consumption

We differ the number of clusters, to verify the effectiveness of our technique. We have chosen 4 configurations.

The first configuration has 2 clusters, and each cluster is composed of one Unit A, one Unit M, and one Unit D. And there are 8 registers in the global register file. Each cluster has 2 read and 1 write access ports to the global register file.

The second configuration has 4 clusters, and each cluster is composed of one Unit A, one Unit M, and one Unit D. And there are 8 registers in the global register file. Each cluster has 2 read and 1 write access ports to the global register file.

The third configuration has 6 clusters, and each cluster is composed of one Unit A, one Unit M, and one Unit D. And there are 16 registers in the global register file. Each cluster has 2 read and 1 write access ports to the global register file.

The fourth configuration has 8 clusters, and each cluster is composed of one Unit A, one Unit M, and one Unit D. And there are 16 registers in the global register file. Each cluster has 2 read and 1 write access ports to the global register file.

**Figure 7:**
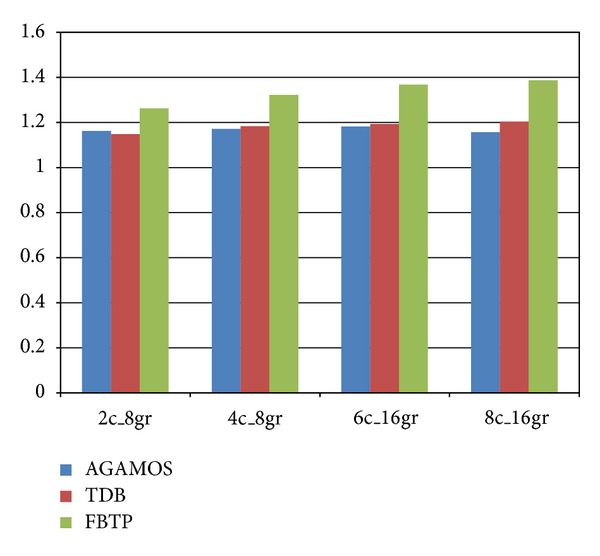
Evaluation of performance with different numbers of clusters.

The result shown in Figure 7 is the performance enhancement compared to the default LilyCC scheduler TDFD. The blue bar represents the performance enhancement of AGAMOS compared to TDFD. The red bar represents the performance enhancement of TDB compared to TDFD. The green bar represents the performance enhancement of FBTP compared to TDFD.

**Figure 8:**
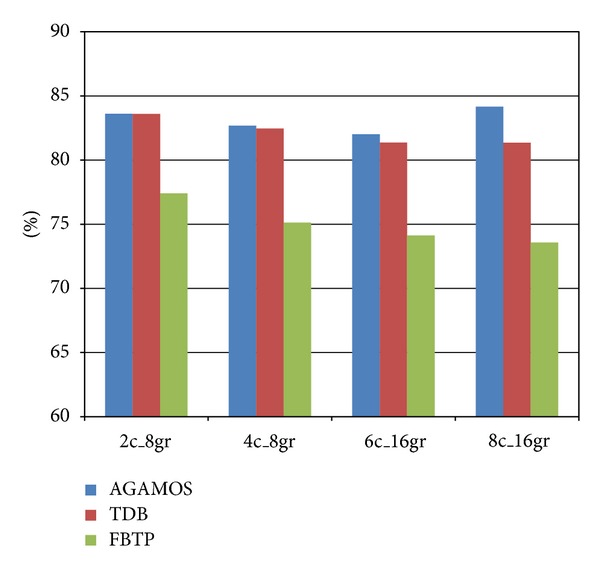
Evaluation of energy consumption with different numbers of clusters.

In all the situations, FBTP outperforms other schedulers. Although AGAMOS and TDB are schedule algorithms optimized for clustered architecture, they are not quite suit for RFCC VLIW. TDFD is designed for RFCC VLIW; however, it simply considered the balancing of influences of data dependence relations and available resources on instruction scheduling but has not actually taken into account the influence of limitation on access ports to the global register file on the instruction scheduling. So, the effectiveness of TDFD on RFCC VLIW is limited. It can be concluded from the figures that the effectiveness of FBTP is not affected by the varying of the number of clusters. The results of energy reduction compared to TDFD are shown in Figure 8.

## 8. Conclusions

In this paper, we have presented an instruction scheduling algorithm for RFCC VLIW architecture which is called FBTP algorithm. FBTP tries to ease the penalty of performance and energy consumption of RFCC VLIW architecture due to limitation of access ports to the global register file. The goal is achieved through (1) dividing the instruction scheduling into two phases, to make decisions of cycle scheduling and cluster assignment in separate phases, but considering the main interactions between cluster assign and cycle scheduling in the process; (2) using gravitation force (GF) value to describe the data dependence relations between instructions, and using repulsion force (RF) value to describe the resource availability; (3) balancing those two forces to conduct the cycle scheduling and cluster assignment, so as to minimize the number of inter-cluster data communications and the number of execution cycles; (4) transforming the distribution of inter-cluster data communications into data dependence relations between instructions and resource availability, when calculating GF value and RF value, in order to minimize the number and scale of concurrent inter-cluster data communications.

We have also presented an LE register allocation mechanism for RFCC VLIW architecture. The LE mechanism is used as an enhancement engine for register allocation in BB, to avoid unnecessary use of global registers, thus to ease the pressure of global register file.

The result shows that our algorithms can largely enhance the performance and reduce the energy consumption. The influence of different types of configure parameters on the effectiveness of the algorithms is evaluated. The performance enhancement compared to default LilyCC scheduler TDFD can up to 38.65%, while the energy consumption reduction compared to default LilyCC scheduler TDFD can up to 26.43%.

## Figures and Tables

**Algorithm 1:**
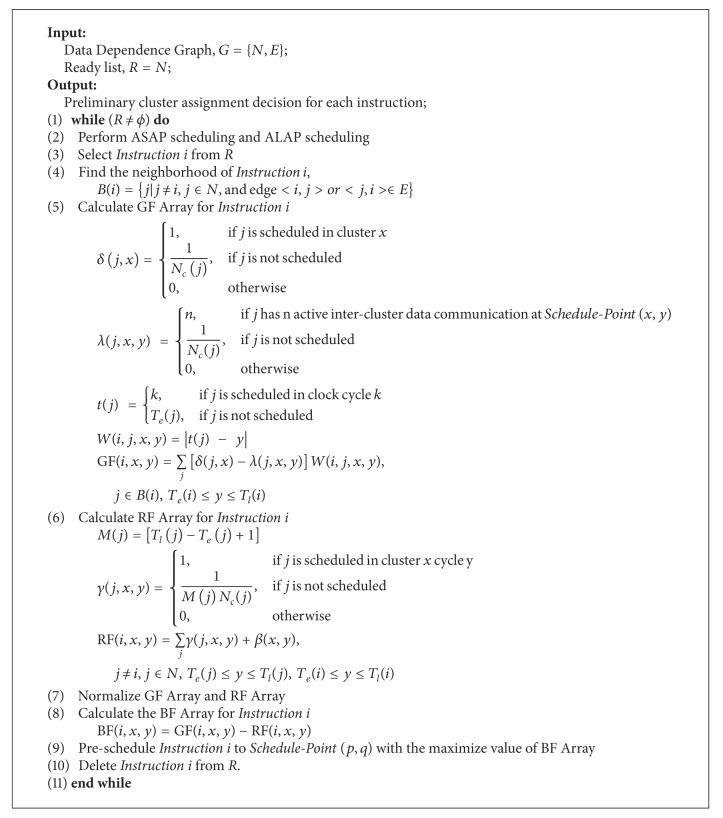
Predecision phase.

**Algorithm 2:**
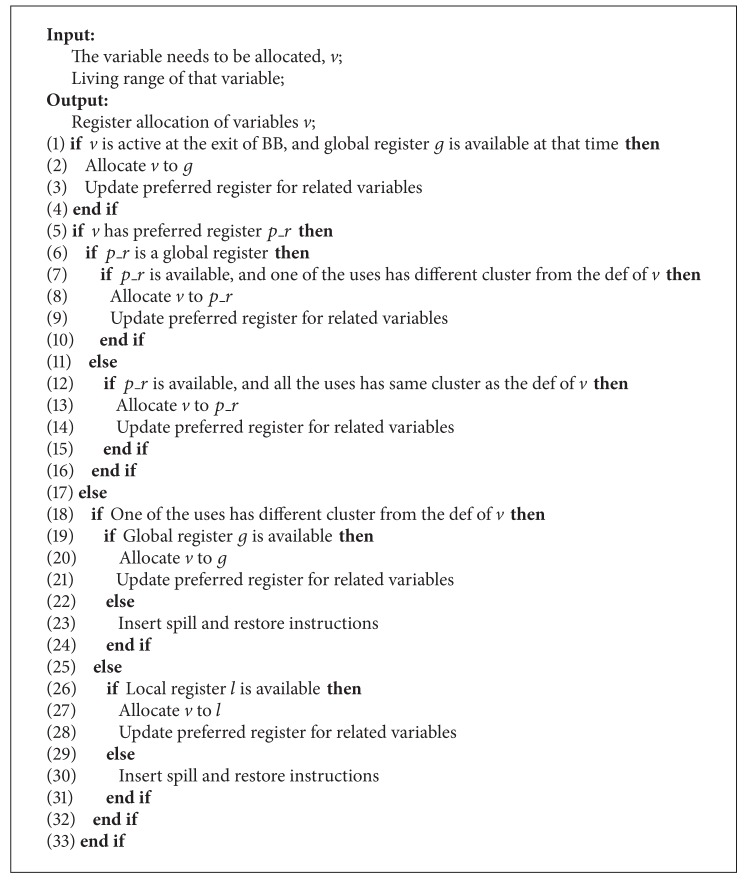
Localization-enhanced register allocation mechanism.
